# Fronto-Central Theta Oscillations Are Related to Oscillations in Saccadic Response Times (SRT): An EEG and Behavioral Data Analysis

**DOI:** 10.1371/journal.pone.0112974

**Published:** 2014-11-18

**Authors:** Adele Diederich, Annette Schomburg, Marieke van Vugt

**Affiliations:** 1 School of Humanities and Social Sciences, Jacobs University Bremen, Bremen, Germany; 2 Dept of Artificial Intelligence and Cognitive Engineering, University of Groningen, Groningen, The Netherlands; Centre de Neuroscience Cognitive, France

## Abstract

The phase reset hypothesis states that the phase of an ongoing neural oscillation, reflecting periodic fluctuations in neural activity between states of high and low excitability, can be shifted by the occurrence of a sensory stimulus so that the phase value become highly constant across trials (Schroeder et al., 2008). From EEG/MEG studies it has been hypothesized that coupled oscillatory activity in primary sensory cortices regulates multi sensory processing (Senkowski et al. 2008). We follow up on a study in which evidence of phase reset was found using a purely behavioral paradigm by including also EEG measures. In this paradigm, presentation of an auditory accessory stimulus was followed by a visual target with a stimulus-onset asynchrony (SOA) across a range from 0 to 404 ms in steps of 4 ms. This fine-grained stimulus presentation allowed us to do a spectral analysis on the mean SRT as a function of the SOA, which revealed distinct peak spectral components within a frequency range of 6 to 11 Hz with a modus of 7 Hz. The EEG analysis showed that the auditory stimulus caused a phase reset in 7-Hz brain oscillations in a widespread set of channels. Moreover, there was a significant difference in the average phase at which the visual target stimulus appeared between slow and fast SRT trials. This effect was evident in three different analyses, and occurred primarily in frontal and central electrodes.

## Introduction

Adaptive behavior depends on the ability of the perceptual system to deliver information about ongoing events in the environment rapidly. This information typically arrives via different sensory channels and has to be integrated to produce a coherent internal representation of the outside world. Recent EEG/MEG studies have shown that input to one sensory modality can reorganize activity in other primary sensory cortices to regulate multisensory processing. That is, neural oscillations reflecting the periodic fluctuations in neuronal activity are reset due to the occurrence of a sensory stimulus (see [1], [2],[3], for a review). In particular, it is assumed that the phase of an ongoing neural oscillation is shifted by the stimulus event so that phase values, even in different sensory modalities, become highly consistent across trials. If two stimuli occur with a certain time lag, the first stimulus would reset an oscillation to its ideal phase; after reset, an input that arrives within the ideal phase evokes amplified responses, whereas inputs arriving during the worst phase are suppressed. For example, somatosensory inputs caused a phase reset of auditory oscillations in monkeys [4], and similarly, visual stimuli could modulate the oscillatory phase of auditory activations [5]. Phase reset tends to occur primarily in the 4–9 Hz theta and 25–55 Hz gamma band [6]. Furthermore, enhanced gamma band oscillations have been observed for crossmodal illusions, for which cross-modal binding is also necessary [7]. In a simple detection study with patients implanted with intracranial electrodes in the context of epilepsy treatment, Mercier and colleagues [8] found auditory-driven phase reset in visual cortices. In particular the theta and alpha bands showed increased phase coherence to audio-visual stimuli relative to audio or visual presented separately.

Evidence of phase reset has also been found in behavioral data using a psychophysical approach. Fiebelkorn and colleagues [9] found that, presenting a sound followed by a near-threshold visual target with stimulus onset asynchrony (SOA), which varied in steps of 500 ms across 6000 ms, that the timing of visual-target presentation relative to the sound influenced the hit rate of visual-target detection. Applying a spectral analysis on the hit rates across the different SOAs, they identified periodicities in the response performance patterns with a frequency lower than 1 Hz.

Diederich and colleagues [10] used saccadic onset times to a visual stimulus preceded by an irrelevant auditory stimulus across a range of 200 ms in steps of 2 ms to probe for underlying oscillatory activity, time-locked to the auditory stimulus. They found that mean response times reductions followed a periodic pattern. Using spectral analysis on the detrended mean response times as a function of SOA they observed performance oscillating in the 20–40 Hz frequency band. Applying a spectral analysis on the trend, they found additional behavioral oscillations between and 7 and 12 Hz.

The phase resetting hypothesis has not only been tested in cross-modal settings but also for (unimodal) entrainment and attention. Assuming that attention operates in a rhythmic manner [11], [12], neuronal oscillations could be the mechanisms behind periodic amplification or attenuation of perceived stimuli [13], [14] since oscillations control neuronal excitability. For example, Varela et al. [15] showed that at some phases of central and parietal alpha oscillations, two brief flashes could be distinguished as two sequential flashes, while at other phases, those were merged into one. Busch et al. [16] showed that also detection of very brief visual flashes was modulated by the phase of 4–8 Hz theta and 8–12 Hz alpha oscillations in frontal channels. Similarly, auditory stimuli that arrive at certain oscillatory phases are better perceived than those arriving at other oscillatory phases [4]. Hanslmayr and colleagues [17] recently found that 7-Hz phase in parietal electrodes prior to stimulus onset predicted performance on a contour integration task. More precisely, during certain phases of the oscillation, there was strong functional connectivity between inferior parietal and occipital regions, which was associated with good performance, while during the opposite phase performance was worse and functional connectivity was reduced.

While all of the above studies focus on the perception side of cognitive tasks, Drew and VanRullen [18] showed that in three different response time tasks, ongoing pre-stimulus activity in fronto-central electrodes in the 11–17 Hz alpha/beta range predicted performance, most likely reflecting improvements in efficiency of response implementation.

Given the observed periodicities in both cognitive performance and brain oscillations in response to cross-modal stimuli, an obvious question is whether there is a relation between the two. And if there is such a relation, what oscillatory frequencies and what brain regions would be involved in this relationship? Given the clear links with attention, an obvious candidate for such a neural substrate is the fronto-parietal attention network [19]. To empirically test this hypothesis, we asked three participants to perform a saccadic response time task in a focussed attention paradigm. The study is a follow-up of the purely behavioral study by Diederich and colleagues [10] using saccadic onset times to a visual stimulus preceded by an irrelevant auditory stimulus across a range of 0 to 404 ms in steps of 4 ms. The increased SOA range relative to that previous study (0–202 ms) allows for detecting periodicities in the response times patterns in the 4–9 Hz theta range. Furthermore, while measuring saccadic onset times, EEG signals were recorded simultaneously. This provides more direct evidence that the auditory accessory resets the neural oscillation phase and helps to better understand how the phase-reset hypothesis manifests itself in behavior.

## Materials and Methods

### Participants

Three students, aged 19 to 26, all female, from Jacobs University served as paid voluntary participants. All had normal or corrected-to-normal vision and two were right-handed (self-description, Coren's Lateral Preference Inventory, 1993). They were screened for their ability to follow the experimental instructions (proper fixation, few blinks during trial, saccades towards visual target). They gave their written informed consent prior to their inclusion in the study and the experiment has been conducted according to the principles expressed in the Declaration of Helsinki. Approval for this study was granted by the Academic Integrity Committee of Jacobs University Bremen.

### Stimuli

The fixation point and the visual stimuli were red light emitting diodes (LEDs) (25 mA, 5.95 mcd and 25 mA, 3.3 mcd, respectively) located on top of the speakers at the same viewing distance of 120 cm, the fixation point in the medial line and the target LEDs 20° to the left and right. Auditory stimuli were bursts of white noise (59 dB(A), rectangle envelope function), generated by two speakers (Canton Plus XS). The speakers were placed at 20° to the left and right of the fixation LED at the height of the participants' ear level and a distance of 120 cm. One PC controlled the stimulus presentation, and two other interlinked PCs controlled the EyeLink program. The control software for the stimulus presentation operated on Realtime-Linux (RTLinux), a hard real-time kernel (RTLinux patched kernel) that runs Linux as its idle thread. Signal output was carried out by a computercard (PCIM DDA06/16), equipped with six digital-analog converters and three digital in- and outports, which fed the control electronic with the generated time signals for the LEDs, the loud speakers and the vibration emitter, the latter not used in the present study.

### Data recording

Eye movements and EEG activity were recorded simultaneously.

Saccadic eye movements were recorded with an infrared video camera system (EyeLink II, SR Research) with a temporal resolution of 500 Hz and horizontal and vertical spatial resolution of 0.01°. Criteria for saccade detection on a trial-by-trial basis were velocity (35°/*s*) and acceleration (9,500°/*s* ^2^). Recorded eye movements were checked for proper fixation at the beginning of the trial, eye blinks, and correct detection of start and end point of the saccade. The proportion of erroneous saccades was less than 2% in most cases (for a detailed analysis of error types, see Table S1). A Brain Vision system (Brain Products) was used to record EEG from 27 electrodes arranged according to the 10/20 system. EEG was amplified by a Brain Vision Amplifier. Data were band-pass filtered from 0.1–1000 Hz in hardware, and from 0.5–250 Hz in software. All impedances were kept below 20 kΩ. All data were referenced to the average of all channels.

The eyetracker system and the EEG system were synchronized by an external trigger signal. The trigger signal was set to 100 ms before the onset of the first stimulus. For the eyetracker system, recording started with the trigger signal and ended 100 ms after the offset of all signals. The EEG signals were recorded in continuous mode and the trigger signals served as markers. Each trial in the EEG signal is windowed in 0.4 s before the trigger (prestim) and 1.5 s after the trigger (poststim section). Data were recorded with a sampling rate of 500 Hz.

### Procedure

The procedure was identical to our previous study [10]. The participants were seated in a completely darkened, sound-attenuated room with the head positioned on a chin rest, elbows and lower arms resting comfortably on a table. Although the eye tracking equipment takes head movements into account, the participants were instructed to leave the head on the chin rest and not to move the head. Prepared with the EEG head cap the participant began every experimental session with 10 minutes of dark adaptation during which the measurement system was adjusted and calibrated. Each trial started with the appearance of the fixation point of random duration (1200–2100 ms). When the fixation LED disappeared, the visual target stimulus was turned on for 500 ms without a gap. Participants were instructed to gaze at the visual target as quickly and as accurately as possible ignoring any auditory non-targets (focused attention paradigm). The visual target appeared alone or in combination with the auditory non-target in either ipsi- or contralateral position.

**Figure 1:**
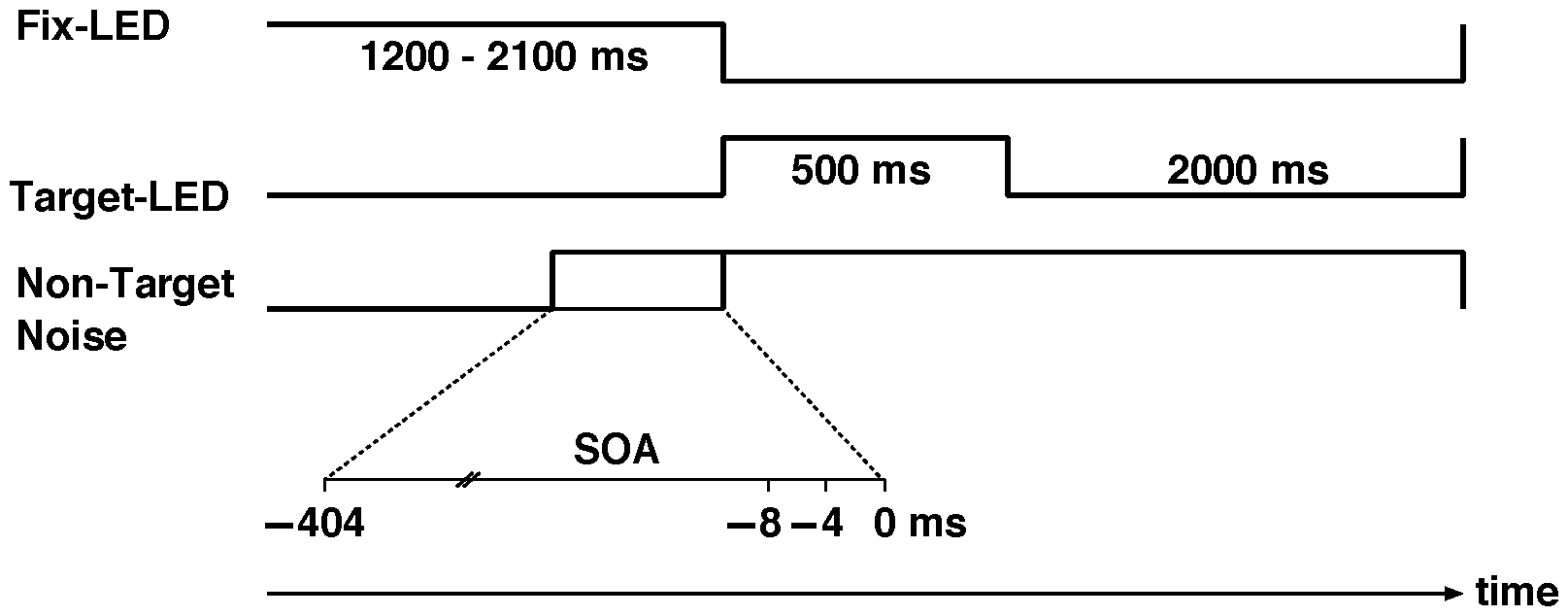
Time course of a trial. First a central fixation light is on, when at some point a sound stimulus is produced. After a variable SOA, the target LED is turned on, to which the participant has to respond whether it is on the left or on the right.

The onset of the auditory non-targets was varied between 404 ms and 0 ms prior to the target in steps of 4 ms, resulting in a total of 102 stimulus onset asynchronies (SOAs) (Figure 1). The non-targets were turned off simultaneously with the visual stimulus. Thus their duration varied between 904 and 500 ms. Stimulus presentation was followed by a break of 2 s in complete darkness, before the next trial began, indicated by the onset of the fixation LED.

One experimental block consisted of 212 trials (204 bimodal, each SOA presented once ipsi- and once contralaterally, 8 unimodal). Trials were randomized over SOA and laterality. Each participant performed a total of 48 experimental blocks–four blocks within one experimental session, which lasted about one hour. Each participant was engaged for about thirteen hours (twelve experimental and one training hour) over the course of several weeks and completed a total of 10,176 experimental trials.

### Data Analysis Saccadic Response Times

For each subject, median saccadic reaction time was analyzed as a discrete time series, considered as a function of the SOA values (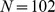), and separately for ipsi- and contralateral presentations. Prior to subjecting the data to a spectral analysis, all time series underwent some preprocessing, as described next.

#### Trend removal

**Figure 2:**
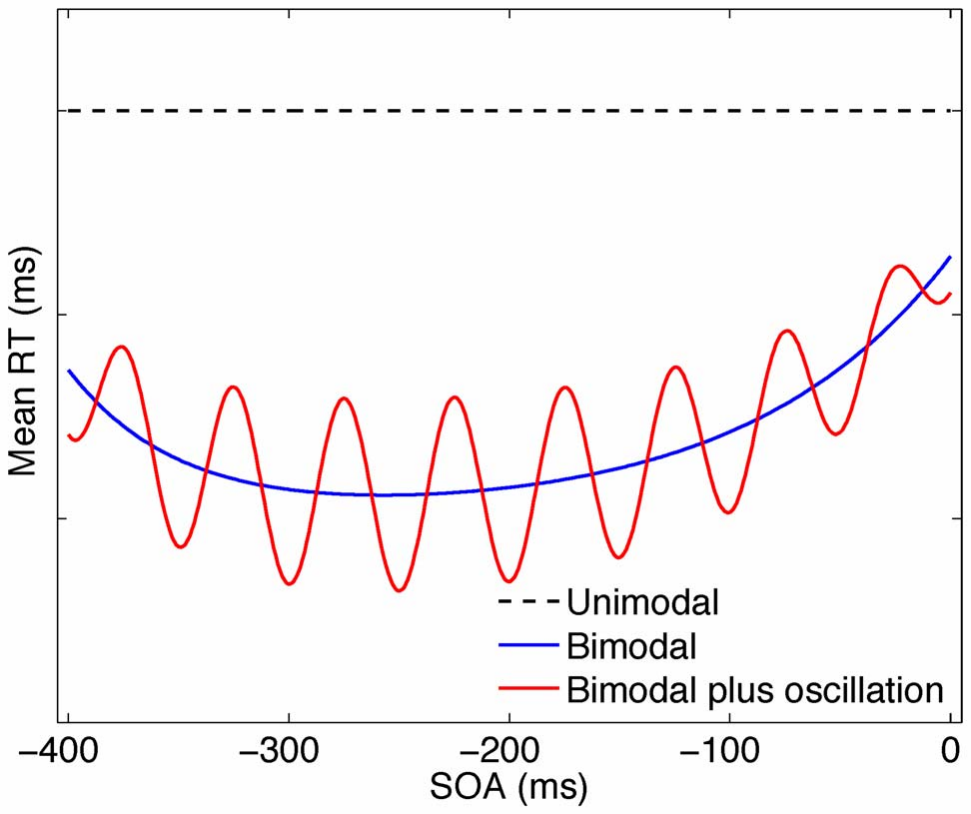
Predictions for mean bimodal RT with and without a phase effect as function of SOA. The black (dashed) line indicates unimodal RT, bimodal (blue/red) line shows mean RT without/with effect of oscillatory activity (idealized functional forms).

It is well known that mean bimodal SRT in a focused attention paradigm exhibits an overall trend with varying SOA: it typically first decreases and then increases with the (leading) nontarget being presented closer and closer in time to the target (see e.g., [20]). The blue line in Figure 2 visualizes these results in an idealized way. For most published experiments only a few SOAs are available (e.g., 0, 50 100, 200 ms) and predicted curves are based on inter- or extrapolation only. The dotted (black) horizontal line indicates mean unimodal SRT to the visual target, providing a benchmark for measuring crossmodal facilitation. The red line illustrates the hypothezised effect of high and low crossmodal excitability, due to resetting, in addition to crossmodal facilitation. Because a trend as indicated in the blue line in Figure 2 can completely nullify the estimation of the frequency spectral content of the signal (Bendat & Piersol, p. 291), it was removed as follows (see also [10]).

Each time series 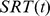 was assumed to be decomposable into two components
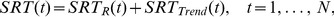
(1)


where 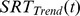 is the trend component to be eliminated and 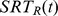, with zero median, contains the remaining constituents of the observed median SRT including oscillation to be subjected to further data analysis. Note that, different from the [10] study we used the median here instead of the mean since that better represents the central tendency of response time distributions.

The trend function was estimated by least-squares fitting of a 5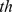-degree polynomial function to 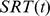 (using MATLAB functions *polyfit* and *polyval*). Note that the polynome was chosen by a stepwise increasing its degree and choosing the first polynomial that provided good visual agreement with the detrended time series. Text S1 and Figure S1 show the effects of using a higher rather than a lower degree of polynomial.

#### Simple Moving Average

The stimuli were presented in SOA steps of 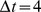 ms, which gives us a sampling rate of 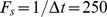 Hz. The largest frequency detectable in the data is then determined by the sampling rate 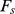, i.e., 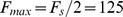 Hz (Nyquist sampling theorem). Because – given the results reported in the EEG studies and single-cell recordings mentioned in the introduction – we are only interested in frequencies below 50 Hz, we applied a simple moving average filter to the time series 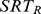 to remove faster fluctuations. Specifically, each point in the filtered time series, 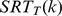, was calculated as 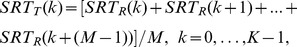
(2)


where 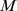 is the filter length. With 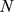 data points in the original data series the filtered data series has 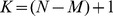 data points. A cut-off frequency of around 50 Hz requires a window length of 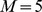 (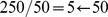
Hz cut-off frequency), resulting in 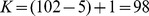 points in the filtered series (SOA: 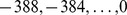) encompassing 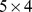
ms 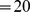
ms.

The smallest frequency that can be detected in the data, i.e., the frequency resolution, is determined by the record length 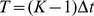. Since the filtered data series has a record length of 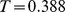
s, the frequency resolution is 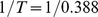
s 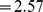 Hz. That is, only frequencies within the range of about 2.5 Hz to 50 Hz are considered here.

The preprocessed discrete SRT time series data, for each subject and for both ipsi- and contralateral presentations, were probed for their spectral components. The power spectrum is a convenient way to show how much of a signal is present at a specific frequency.

#### Power spectrum

On the filtered, zero-median data series 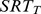 we performed a spectral analysis to separate data series into different periodic components. Note that this technique is purely descriptive to discover cyclical phenomena. The Discrete Fourier Transform (DFT) decomposes 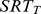, the input signal in the time domain, into an output signal in the frequency domain 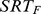, containing estimates of the amplitude and phase of the sinusoidal components. The DFT was carried out by MATLAB function *dft* using a zero padding methods. That is, the time series was padded with zeros to increase the number of sampling points from 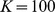 to 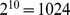 sampling points. Thereby, the frequency resolution was enhanced from 2.5 Hz to 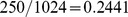 Hz. The absolute value (magnitude) of the Fourier coefficients represents the amplitude of the spectral components, with its square as the power spectrum. This reflects how much periodicity is visible in SRTs at each particular frequency.

#### Statistical Tests

To test for the possibility of artifacts due to the antecedent numerical procedures we performed the same analyses as on the original data but under random permutations of the time points. If the spectral analysis results of the original data are not significantly different from those under random permutations of the time points, then our hypothesis of an oscillatory activity in response times would not be supported by the observed data. Specifically, we first considered how the amplitude of the frequency component that was maximal in the original time series was distributed across the power spectra generated from 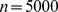 shuffled time series that were randomly drawn from the set of all 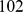 possible permutations. However, because frequency resolution is limited to about 2.5 Hz, the spectra from the DFTs on the shuffled data may not contain power at the exact maximum frequency. Therefore, the amplitude at the maximum frequency was merged with the amplitudes occurring for 10 evenly-spaced frequency levels around it within a 2.5 Hz range.

As an additional test, we compared the spectrum of the original time series to the average spectrum across all 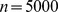 shuffled time series.

### Data Analysis EEG data

#### Artifact correction

Data analyses were performed with the help of the Fieldtrip toolbox [21]. We first removed artifacts by visual inspection, which removed 25.2%, 11.6%, and 14.0% of trials for participants 1, 2, and 3, respectively. This was followed by ICA decomposition to remove eye blinks and muscle activity. Finally, 50 Hz line noise was removed with a bandstop filter.

#### Phase computations

For each correct trial the instantaneous phase 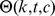 of the EEG-signal was calculated by *Hilbert Transform*. Hilbert transforms have previously been shown to give the most reliable phase estimates [22], [23]. Here *t* represents the time sample within trial *k* for channel **c**. A small bandpass filter was used to extract the frequencies in the range observed in the behavioral data. The filter range was set to 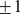 Hz of the center frequency.

#### Statistical tests

We then asked for each channel whether there was significant phase locking just after the sound using a Rayleigh test. These tests were done for every participant individually. To then assess what channels showed both significant phase-locking to the sound (relative to a pre-sound baseline) and a significant difference in phase between slow and fast RTs, we used randomization tests with 200 iterations, done for every participant individually. To examine the significance of phase locking, we randomly assigned data points to baseline and sound intervals, and recomputed the phase locking statistic to the sound. We compared the empirically observed phase locking to the sound to this randomized phase locking statistic, and turned this probability into a 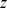-score.

In addition, we examined whether there was a significant difference in phase between the shortest and the longest half of the response times using a Watson-Williams test for equality of circular means. In the randomization test of this analysis, we permuted the short and long RTs and recomputed the phase difference. We compared the empirically-observed to the "random'' phase difference, and converted the final probability into a z-score. We further examined the phase-specificity of the RT effect with the phase bifurcation index 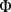 developed by [16]. This phase bifurcation index compares the phase distributions for two conditions (in this case, the short and long RTs; see equation 3). When the phases are locked to different phase angles for long and short RTs, then 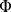 will be positive. When 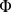 is 1, this indicates perfect phase-locking in both conditions to opposite angles; when the two conditions have random phase angles, 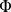 is 0. When only one of the conditions exhibits phase locking, then 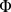 becomes negative. 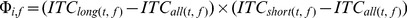
(3)


As a last measure of whether RT depends on pre-stimulus phase, we regressed RT directly on the phase just before the light appeared on the screen. We used a circular-linear correlation measure to perform this regression.

To examine the specificity of the results across frequencies, we repeated the statistical tests for phase locking and phase differences for a set of logarithmically-space frequencies, and graphed the statistics averaged across participants.

## Results

### Data screening

Saccades were screened for anticipation errors (SRT 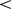 80 ms), misses (SRT 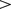 500 ms), and accuracy: trials with saccade amplitude deviating more than three standard deviations from the mean amplitude were excluded from the analysis. Table S1 lists the percentages of different error types for each participant. The error rates are very low throughout. There was no evidence for multiple saccades in the remaining data set.

### Crossmodal Facilitation of Saccadic Reaction Time

**Figure 3:**
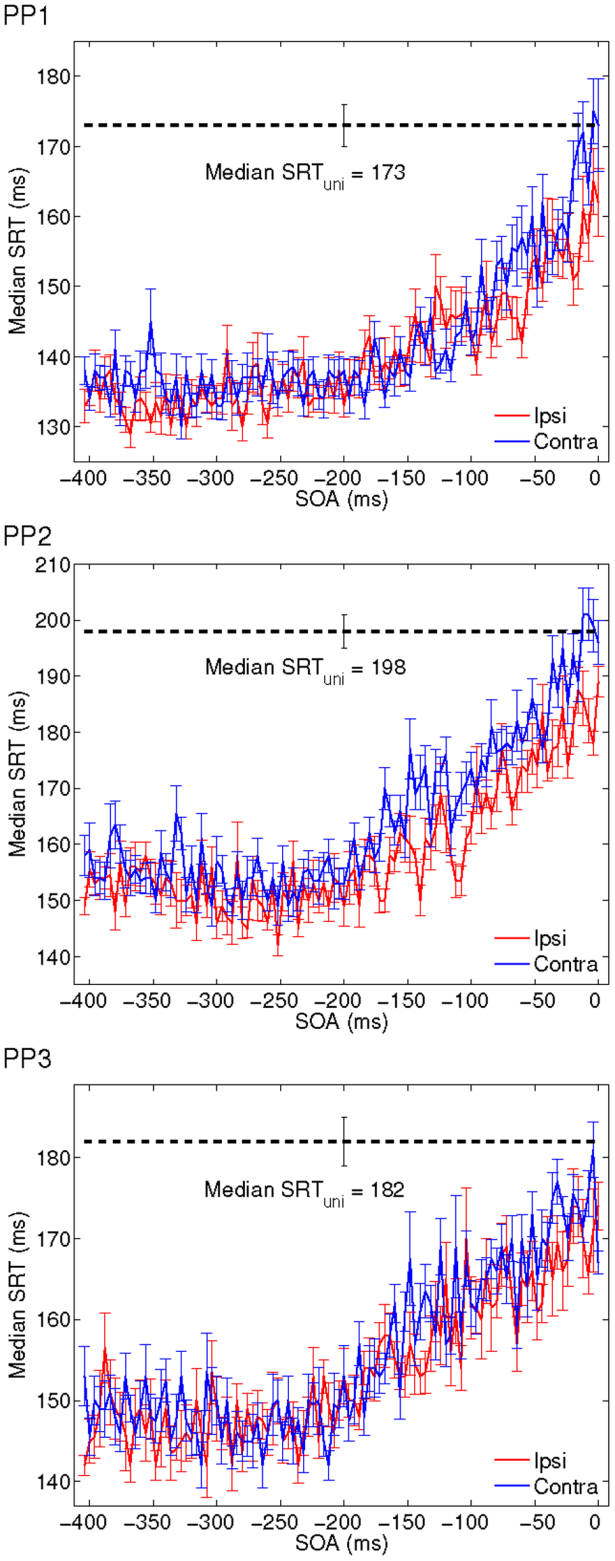
Observed median SRT (±2 standard errors) as a function of SOA for all participants. Unimodal median SRTs are indicated by the dotted line including the error bar.

Figure 3 shows median saccadic response times to unimodal and to bimodal as a function of SOA for all participants, including error bars. The error bars indicate 95% confidence intervals (1.58 quantile). Median SRTs to bimodal stimuli are shorter than to the unimodal stimuli for all participants except for very short SOAs. Specifically, responses tend to speed up with the (leading) auditory nontarget being presented earlier relative to the visual target, and P2 and P3 exhibit a typically observed spatial effect, i.e., faster responses to the ipsilateral configuration for shorter SOA (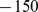 to 0 ms). Note that all graphs show a considerable fluctuation of mean SRT from one value of SOA to the next.

To quantify the observed amount of facilitation we calculated a measure of multisensory response enhancement (*MRE*), which compares mean SRT in the bimodal conditions to that in the unimodal condition [24].

The larger this number, the more SRT benefits from seeing a multimodal rather than a unimodal stimulus. 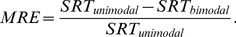
(4)


A summary, showing the minimum, maximum, mean and median relative amount of facilitation across all SOAs for each participant separately, is provided in Table 1. A negative value indicates inhibition rather than enhancement due to adding in a second stimulus modality.

**Table 1:** Multisensory response enhancement.

Participant	MRE for Bimodal Stimuli presented					
	Ipsilateral			Contralateral		
	Max	Min	Mean (median)	Max	Min	Mean (median)
1	27	3	18 (20)	24	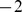	17 (20)
2	30	1	21 (23)	30	 1	18 (21)
3	24	5	16 (17)	22	 3	15 (17)

Minimum and maximum amount of multisensory response enhancement (MRE) in % for ipsi- and contralateral stimulus presentations (across all SOA values).

### Spectral Analyses on Behavioral Data

To quantify periodic fluctuations in the behavioral data, we performed spectral analyses.

#### Power spectrum and statistical test

**Figure 4:**
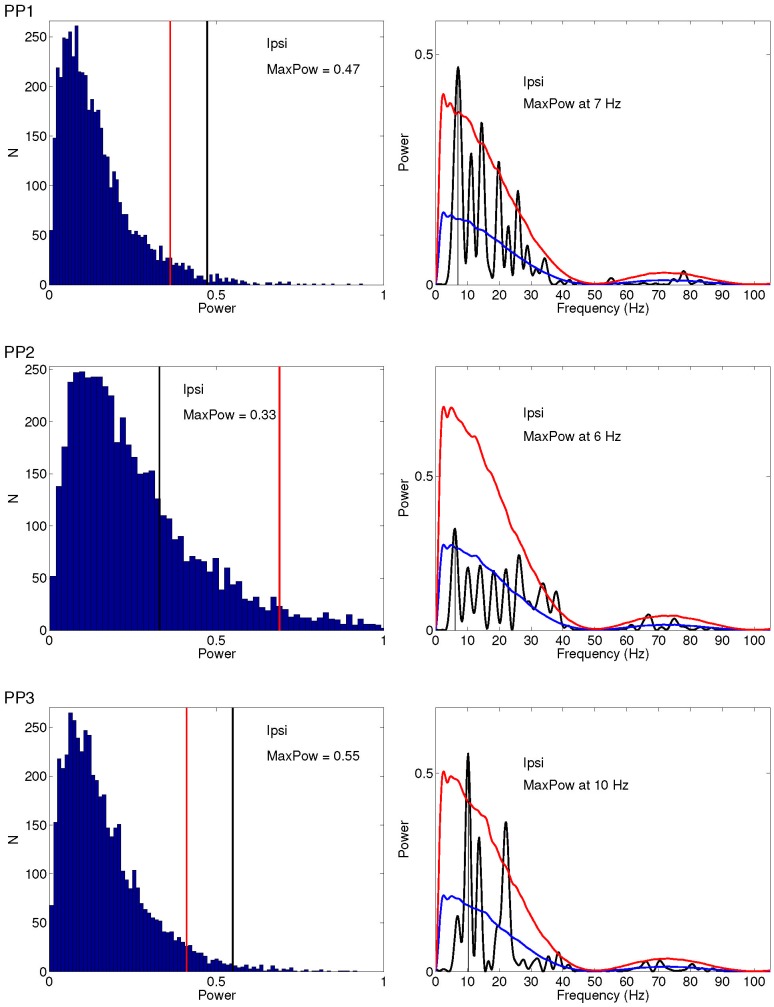
Statistical significance of periodicity in SRTs for ipsilateral presentation for all participants. Left: Distribution of amplitudes across shuffled time series (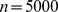) of the frequency that showed maximum amplitude in the observed time series Right: The original spectrum (black line) plotted against mean spectrum (blue line averaged across n = 5000 spectral samples from the set of shuffled time series. Red lines indicate one-sided confidence interval bound (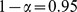).

**Figure 5:**
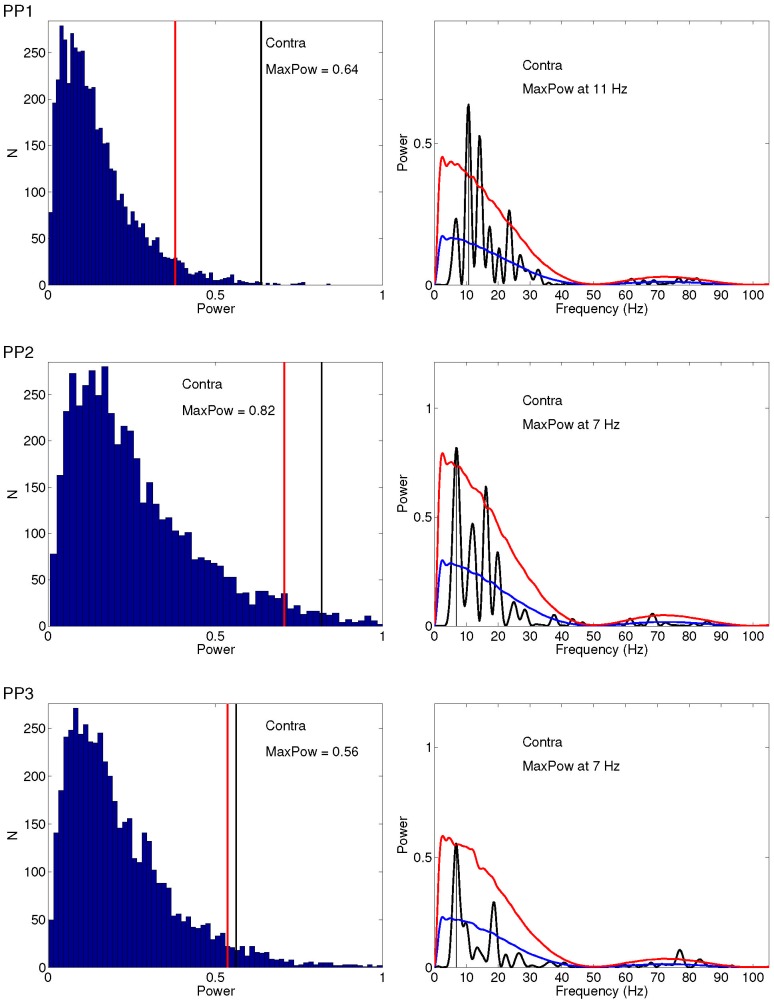
Statistical significance of periodicity in SRTs for contralateral presentation for all participants. Left: Distribution of amplitudes across shuffled time series (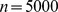) of the frequency that showed maximum amplitude in the observed time series Right: The original spectrum (black line) plotted against mean spectrum (blue line averaged across n = 5000 spectral samples from the set of shuffled time series. Red lines indicate one-sided confidence interval bound (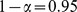).

Distinct peak spectral components can be observed for both spatial conditions across all participants. For all participants maximum power is observed primarily between 6 and 11 Hz, equivalent to an oscillation with period lengths of 91 to 167 ms. Depicting the resulting distribution of amplitudes, Figures 4 and 5, left panels, show that the amplitude of the peak frequency in the observed time series is significantly larger than those from the shuffled time series in five out of six cases: for participant 1 and participant 3 for both conditions, for participant 2 for the contralateral condition. The vertical red line indicates the the maximum power value that is surpassed by 5% of the bootstrap sample values, whereas the black vertical line indicates the maximal power observed in the data. Figures 4 and 5, right panels, depict the average spectrum of the shuffled time series with the corresponding (one-sided, 95%) confidence bound calculated from the original spectrum for ipsi- and contralateral presentation, respectively. One may wonder how sensitive those results are to the degree of the polynomial. Text S2, Table S2, and Figures S2, S3 show the results when using a 2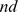-degree polynomial for the detrending, replicating periodic fluctuations in the theta band. In addition, an analysis of the 5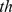 degree trend itself for periodic fluctuations shows that the phenomena we observe are not an artifact of the detrending procedure.

One of the reviewer's requests was to use the lower order polynomial for detrending. We have shown in Table S2 how the order of the polynomial affects the peak frequency of the oscillations in response time. Specifically, this table shows that for three out of six cases, results are identical between polynomials of orders 2 and 5. For the remaining three cases, the peak frequency shifts to 3 or 4 Hz instead of 7 to 11 Hz. However, there are at least two reasons to consider such low frequencies to be irrelevant: 1) Electrophysiological studies of the effects of oscillatory phase on perception have thus far shown that only oscillations in the 5–16 Hz theta/alpha range are relevant (see e.g., [16], [18], [25]. We therefore think that the 3 Hz oscillation in response times is driven by the frequency of the task in general, and does not lead to specific oscillatory phase reset. 2) More importantly, mean response time in our saccadic response time task is known to fall off to a minimum SOA of around 150 ms (see Figure 3 and also previous work on intersensory facilitation). Transforming this into a frequency leads to approximately 3–4 Hz. This general fall-off of response time with SOA is exactly what our trend analysis is designed to pick up. If that frequency is not removed from the behavioral data, we would be focusing on the general fall-off with time, rather than the super-imposed behavioral oscillations that we are interested in (see also [10]). Taken together, this suggests that a polynomial of order 5 is better suited for detrending the behavioral data than a polynomial of order 2.

### Phase reset of EEG by first stimulus

**Figure 6:**
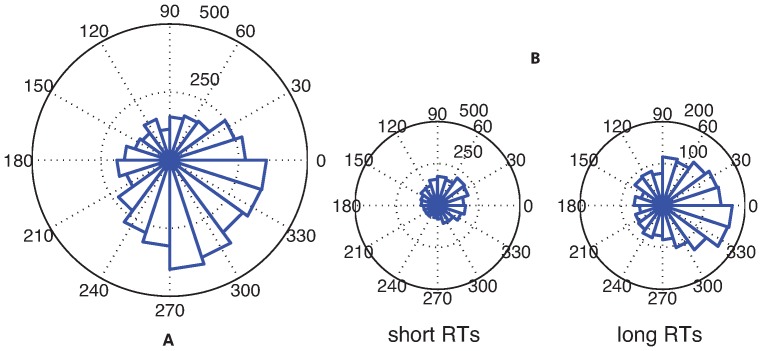
EEG phase reset effects. Phase reset by auditory stimulus (a) and differences in phase between long and short saccadic RTs (b). Phase effects are shown for participant 3, but other participants show similar results.

The behavioral analyses indicate that for every participant the oscillatory frequency in SRTs at which intersensory facilitation is maximized is 7 Hz. If these oscillations in behavior are associated with oscillations in the EEG, the mechanism through which this could occur is phase reset. Specifically, the first (sound) stimulus should reset on-going oscillations, which then can cause the second (light) stimulus to appear at either a more favorable or less favorable phase of ongoing oscillations, depending on SOA (Figure 6).

**Figure 7:**
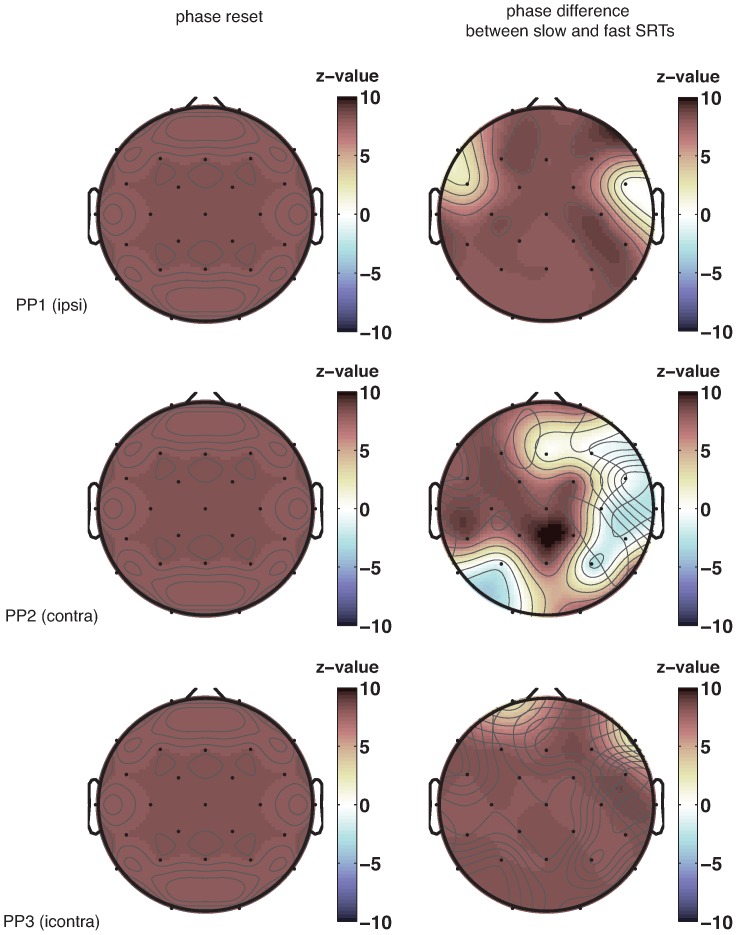
Randomization tests of phase-locking. Topographical plots of z-scores of the randomization tests of phase-locking to the sound (1) and phase-dependent differences in SRTs (2) at the frequency of 7 Hz. Every row shows a different participant. Phase reset due to the sound stimulus occurs in almost all channels. A phase difference between fast and slow SRTs occurs primarily in central channels.

**Figure 8:**
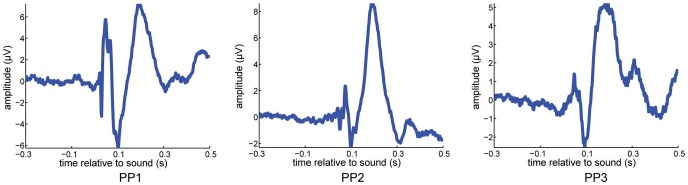
Evoked potentials in relation to sounds. Every column shows a different participant, and time  =  0 reflects the delivery of the sound stimulus. The sound stimulus clearly resets the EEG and creates an evoked potential.

We therefore computed phase consistency of 7-Hz EEG oscillations at the time of the sound and compared that to phase consistency just prior to the sound. The left column in Figure 7 shows topographical plots of the channels that exhibit a significant difference in phase locking between time points just before the appearance of the sound, and time points just after that. Phase locking of 7 Hz oscillations to the presented stimulus occurs in the whole brain. In addition, simple event-related potentials (Figure 8) exhibit clear evidence of evoked potentials due to the sound stimulus.

### EEG phase differences between more and less facilitated SRTs

Having observed that the sound stimuli do indeed reset the phase of on-going oscillations – as we had predicted – we next investigated whether such reset also has consequences for SRTs. In particular, after having corrected for the general decrease in SRT with SOA using the polynomial fit, there should be a difference in the EEG phase at which the light stimulus appears for fast and slow SRTs (Figure 6). In other words: if the oscillation in SRT is caused by the light appearing at a favorable or unfavorable phase of the on-going oscillation, then there should be a difference in oscillatory phase between relatively fast and relatively slow SRTs (in the Figure, slow SRTs have a preferred phase around 30 degrees, while fast SRTs have a preferred phase of 340 degrees). The right column in Figure 7 indicates the channels for each participant that show a significant phase difference at the onset of the light between relatively fast and relatively slow SRTs.

A plausible neural correlate of the observed oscillations in SRTs should show evidence for both a phase reset, and a difference in phase between fast and slow SRTs. Across our participants, a set of channels in central regions shows this pattern. Figure 6 illustrates these effects for a single channel (central channel C3). There is a significant phase uniformity in response to the sound stimulus. In addition, the phase distribution between fast and slow SRTs differs, with a different peak in the histogram of phase angles for faster and slower SRTs. However, the Watson-Williams test used here presupposes that there is significant phase-locking, which does not seem to be the case here. We therefore decided to use additional measures of the same phenomenon.

**Figure 9:**
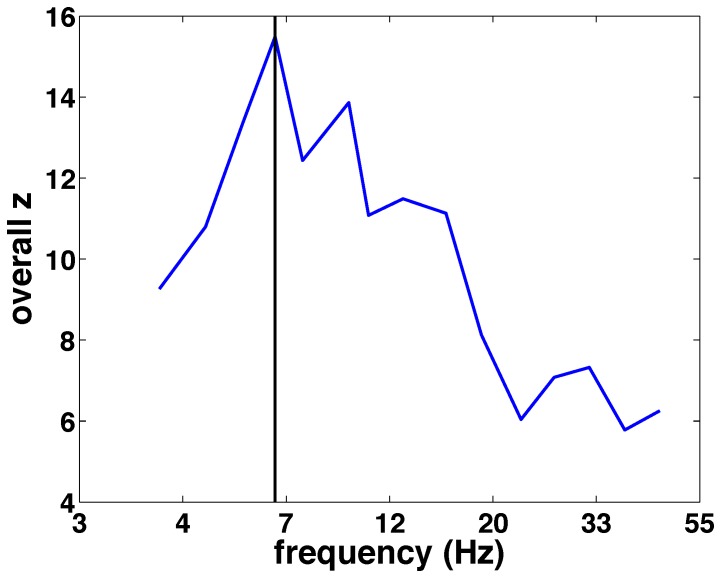
Channels showing a significant circular-linear correlation between phase at which the light appears and saccadic response time. Colors indicate all channels that have a correlation with a p-value smaller than 0.05, where darker colors reflect stronger correlations. Largest circular-linear correlations arise in fronto-central channels.

An alternative way to measure whether pre-stimulus phase of the light depends on SRT is to ask whether SRT depends on phase using a circular-to-linear correlation. Figure 9 demonstrates that there is a small but significant (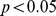) circular-to-linear correlation between pre-stimulus phase and response time in central channels. A drawback of this analysis, however, is that the relationship between phase and SRT is not linear. It is therefore a good idea to investigate yet another measure of the relationship between RT and pre-stimulus phase.

**Figure 10:**
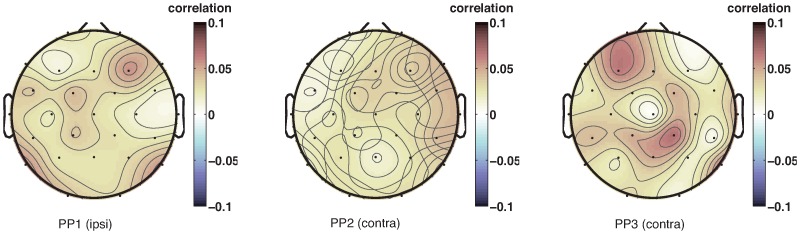
Channels showing a significant phase bifurcation index comparing the relatively short and relatively long SRTs. All colored channels have a p-value smaller than 0.05, and darker colors indicate a larger difference between average phases and/or stronger phase locking. Largest phase bifurcation index occurs in fronto-central channels.

A third way to measure whether pre-stimulus oscillatory activity affects SRT is the phase bifurcation index developed by [16]. This method compares the amount of phase locking between two conditions, relative to the conjunction of both conditions. Figure 10 shows that similar to the circular correlation analysis, a set of fronto-central channels shows a significant (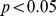) difference in average phase between the faster and slower SRTs. In contrast to the previous method, this method is very sensitive to the amount of phase-locking, such that the results are very weak in cases where the overall locking to a specific phase is low (which is true in our case).

While all three methods have their drawbacks, together they indicate there is evidence for an effect of pre-stimulus phase on RT in a stimulus detection task, taking place primarily in frontal and central channels.

**Figure 11:**
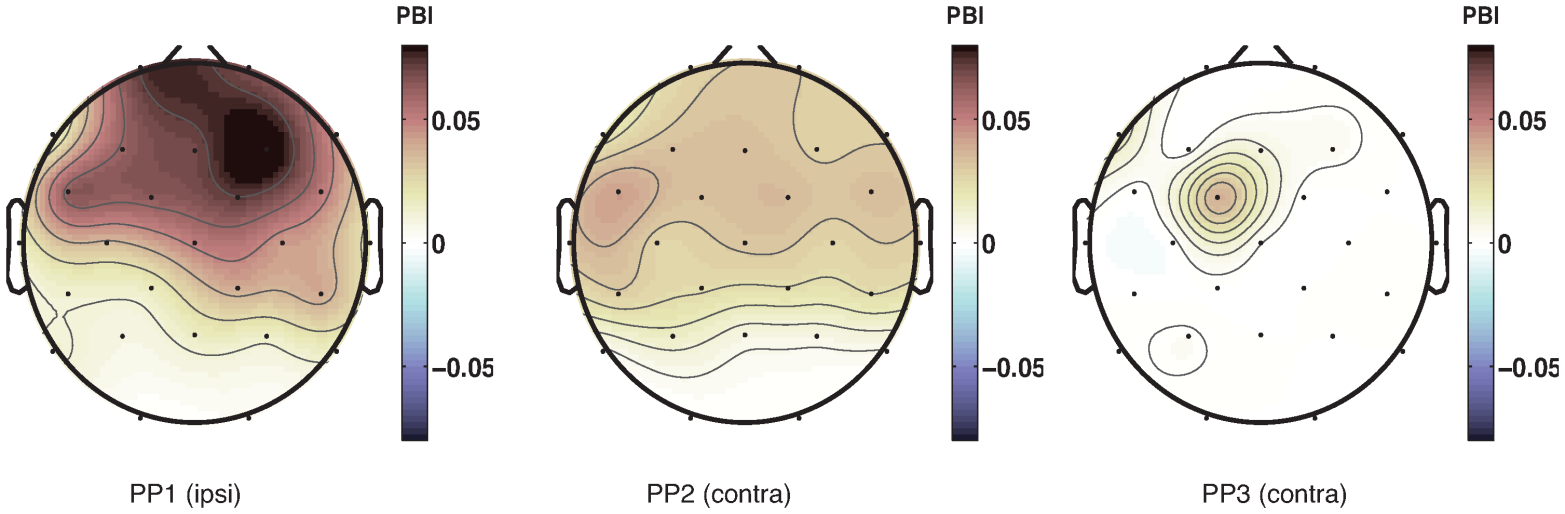
Frequency specificity of the combined phase-locking and phase-difference effects. Average significance of the phase-locking to the sound and phase difference between short and long saccadic response times. Maximum phase-locking/phase difference effects arise at 7 Hz, the frequency at which behavior is also modulated.

Having established the presence of a phase difference between short and long RTs, we examined how specific the effect was to 7 Hz, which is the oscillation that emerged from the participants' behavior. We redid the Rayleigh and Watson-Williams tests for a series of logarithmically-spaced frequencies. Figure 11 shows that indeed 7 Hz is the frequency with the most significant effects of both phase-locking and phase-dependent RT facilitation. Furthermore, these effects are robust to method of phase determination; Text S3 and Figures S4–S8 show that qualitatively similar results are obtained when measuring oscillatory phase with wavelets rather than the Hilbert transform.

## Discussion

The phase-reset hypothesis for multisensory integration holds that crossmodal interaction is evoked by the occurrence of a sensory stimulus shifting the phase of an ongoing neural oscillation to a specific value such that the processing of a subsequent stimulus in another modality is either suppressed or facilitated, depending on the exact relation between the phase of the neural oscillatory activity and occurrence of the second stimulus.

In a follow up study to Diederich et al. [10], we presented a supra-threshold auditory accessory stimulus (non-target) followed by a visual target stimulus at a specific stimulus onset asynchrony (SOA). The current study differs from our earlier work in two important ways. First, the range of SOAs was doubled, i.e., its presentations varied randomly between 0 and 404 ms in steps of 4 ms. This allowed us to detect lower frequencies that include the theta range. Second, in addition to saccadic onset times we simultaneously measured EEG signals that could provide direct evidence for the auditory accessory stimulus resetting the neural oscillation phase, with corresponding consequences for the visual target stimulus. It also allowed us to test whether the observed behavioral oscillations were accompanied by corresponding oscillations in the brain. Through this, we will be able to better understand the contribution of behavioral data to the phase-reset hypothesis.

Similar to our previous study [10], mean/median saccadic reaction time (SRT) to the crossmodal stimulus exhibited a speedup of responses (facilitation) of up to 70 ms compared to responses to the unimodal visual stimuli. This corresponds to a multisensory response enhancement up to 30% (Eq. 4).

Using discrete Fourier analysis on the detrended and smoothed times series (mean SRT indexed by SOA), we observed distinct peak spectral components in the power spectra within a frequency range of 6 to 11 Hz, and with a modus of 7 Hz across ipsi- and contralateral presentation. Subsequent statistical tests, comparing the observed results with those obtained from random shuffling of the time points, supported the significance of the observed peaks in five out of six instances. In our previous study [10], the significant speaks could be found between 20 and 40 Hz due to the shorter SOA range of 200 ms. Interestingly, however, a spectral analysis of the trend component, 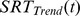, which was eliminated from the time series, 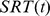, (Eq. 2) showed maximal power between 7 Hz and 12 Hz, with a modus of 8 Hz (based on 12 power spectra, [10], supplementary information).

The EEG analysis showed how the auditory accessory stimulus, presented first, caused a phase reset in 7-Hz brain oscillations in a widespread set of channels. Moreover, there was a significant difference in the average oscillatory phase at which the visual target stimulus – presented second – appeared between slow and fast SRT trials. This effect showed up in three different analyses, and occurred primarily in frontal and central electrodes. Most interestingly, the effect occurred specifically at the 7 Hz frequency that manifested also in participants' behavior.

Our results are in line with a number of recent studies also investigating the phase resetting hypothesis. In a combined response time-EEG study with healthy participants Thorne and colleagues [26] showed that visual input resets activity in the auditory cortex. In a discrimination task using short audiovisual stimulus streams they analysed both the response time to the initial stimulus in the stream and to the target stimulus (visual or auditory) to test the phase resetting hypothesis. They found evidence for greater phase resetting with shorter response times.

Romei and colleagues [27] presented brief sounds followed by a occipital transcranial magnetic stimulation (TMS) across a SOA range from 30 to 300 ms in steps of 15 ms to measure visual cortex excitability (phosphene perception rate). Concurrently they recorded electroencephalography. Phosphene perception rate against time postsound showed a periodic pattern with a frequency of about 10 Hz phase-aligned to the sound; this periodicity could also be observed in the EEG data.

Investigating attentional selection mechanisms, Fiebelkorn et al. [28] measured change detection of a near-threshold visual target at a function of different cue-to-target intervals randomly from 300 to 1100 ms. They employed three different conditions: detection at a cued location(spatial selection), detection at an uncued location within the same object (object-based selection) and detection at an uncured location within a different object (absence of spatial and object-based selection). To estimate the time course of visual-target detection, they calculated location-specific detection rates within 50 ms bins and moved them by a window of 10 ms, and performed a fast Fourier transform on the detrended behavioral time-series data similar to the present study. A non-parametrical statistical test revealed significant peaks at about 8 Hz for the cued and same-object locations. in contrast, for the different-object location condition they observed periodicity at 4 Hz. They concluded that there is a moment-to moment reweighing of attentional properties based on object properties and that this reweighing occurs through periodic patterns within (at 8 Hz) and between (at 4 Hz) objects.

Although we believe that the exact frequency is of minor importance, here it corresponds to those found by Fiebelkorn and colleagues [28], who did an analysis of behavioral data, and of Busch and colleagues [16] and Hanslmayr and colleagues[17] for electrophysiological data. The observed difference we observe between average phase for slow and faster SRTs is relatively small, but we think this results from the generally-low phase concentration to the weak visual stimulus and the combined noise of behavioral and electrophysiological measurements. Yet, the fact that the effect can be observed in three different analyses, and is specific to the frequency also observed in behavior strengthens the link to the behavioral effect.

The combined SRT-EEG study gives further support for the idea that the phase-reset hypothesis plays a major part in multisensory integration. Furthermore, it is the first study to show how oscillations in SRTs for visual-auditory stimuli may arise, by means of combined SRT and EEG data analysis. This may also shed some light on methodological issues raised by [29] when determining the phase reset in humans using electrophysiological data. They argue that technical issues like the appropriate use of filters do not arise in behavioral approaches as "periodicity in the response profile provide good prime facia indication of phase effects." (p.148) Since the analysis of both the EEG data and the SRT data revealed the same frequency our findings provide strong support for the phase-reset hypothesis.

## Supporting Information

Figure S1 **Zeromeans after detrending.** Zeromeans after detrending the original time series with a 2*nd* (A) and 5*th* (B) degree polynomial.
(TIFF)

Figure S2 **Procedure for determining lower frequencies.** The observed median SRT with its trend function, a polynomial of degree 5 (left upper panel); the trend (black) of the trend function (red), a polynomial of degree 2 (upper right); the difference between both trend functions, zeromedian difference function (lower left); power spectrum of the zeromedian difference function (lower right.).
(EPS)

Figure S3
**Statistical significance of periodicity in SRTs for ipsi- and contralateral presentation for all participants.** The test was performed on the 2*nd* degree polynomial detrended times series. The original spectrum (black line) plotted against mean spectrum (blue line averaged across n = 1000 spectral samples from the set of shuffled time series. Red lines indicate one-sided confidence interval bound (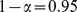).(TIFF)

Figure S4 **EEG phase reset effects.** Phase reset by auditory stimulus (a) and differences in phase between long and short saccadic RTs (b). Phase effects are shown for participant 3, but other participants show similar results.
(EPS)

Figure S5 **Frequency specificity of the combined phase-locking and phase-difference effects.** Average significance of the phase-locking to the sound and phase difference between short and long saccadic response times. There is a clear peak at the frequency of 7 Hz, which also shows the clearest behavioral effect.
(EPS)

Figure S6 **Randomization tests of phase-locking.** Topographical plots of z-scores of the randomization tests of phase-locking to the sound (1) and phase-dependent differences in SRTs (2) at the frequency of 7 Hz. Every row shows a different participant. Almost all channels show significant theta phase reset by the sound stimulus. For most participants, central channels show the largest difference between fast and slow SRTs.
(EPS)

Figure S7 **Channels showing a significant circular-linear correlation between phase at which the light appears and saccadic response time.** Colors indicate all channels that have a correlation with a p-value smaller than 0.05, where darker colors reflect stronger correlations. Significant correlations occur primarily in central channels.
(EPS)

Figure S8 **Channels showing a significant phase bifurcation index comparing the relatively short and relatively long SRTs.** All colored channels have a p-value smaller than 0.05, and darker colors indicate a larger difference between average phases and/or stronger phase locking. A significant phase bifurcation index is observed primarily in central channels.
(EPS)

Table S1 **Percentage of errors by type for each participant.**
(PDF)

Table S2 **Dependence of frequencies with maximum power on order of detrending polynomial.** Frequencies with maximum power after detrending the median data series with polynomials of two different degrees.
(PDF)

Text S1 **Detrending with different degree polynomials.**
(PDF)

Text S2 **Analysis on trends.**
(PDF)

Text S3 **Wavelet analysis.**
(PDF)
