# Supplementary figures and images for: Fronto-Central Theta Oscillations Are Related to Oscillations in Saccadic Response Times (SRT): An EEG and Behavioral Data Analysis
Source: PLoS One. 2014 Nov 18;9(11):e112974. doi: 10.1371/journal.pone.0112974 (PMC4236144; doi:10.1371/journal.pone.0112974)

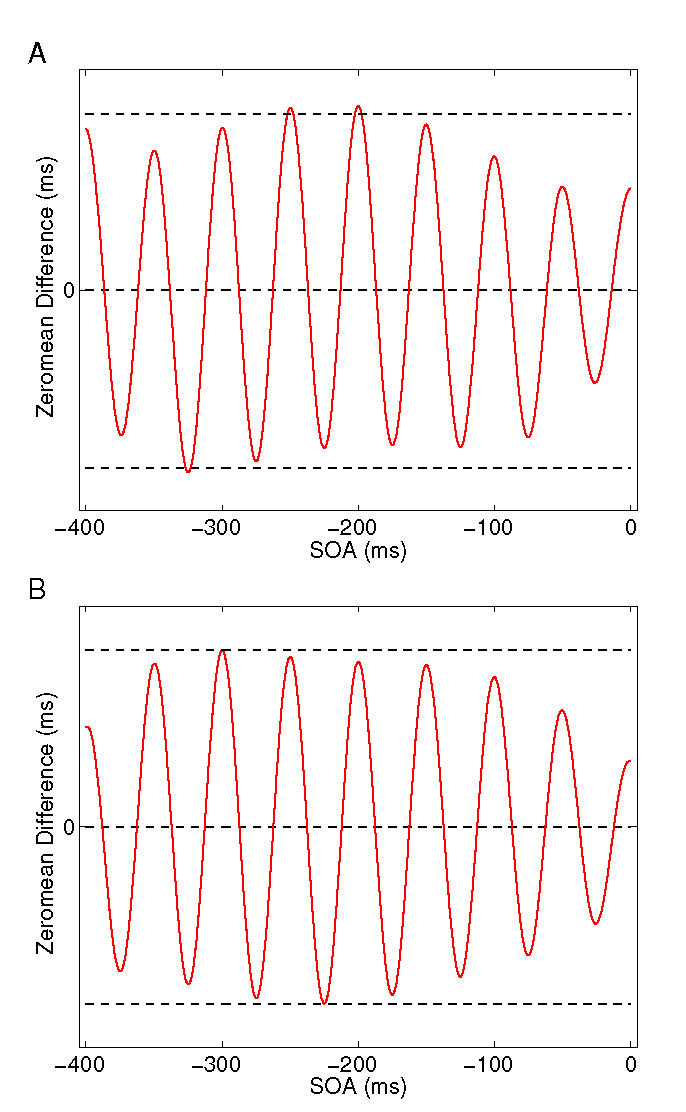

Supplement: Figure S1 — Zeromeans after detrending. Zeromeans after detrending the original time series with a 2nd (A) and 5th (B) degree polynomial. (TIFF) [file pone.0112974.s001.tiff]

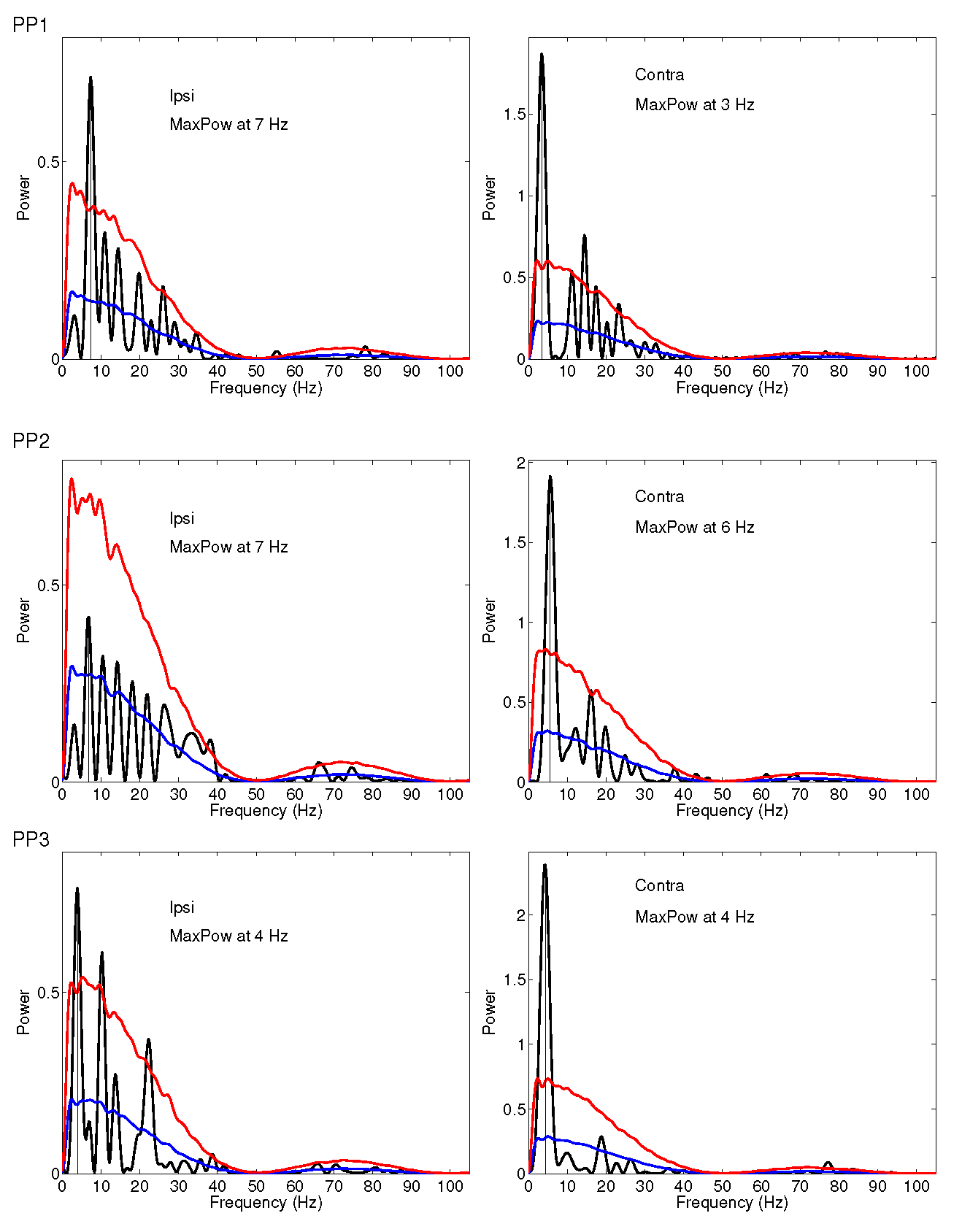

Supplement: Figure S3 — Statistical significance of periodicity in SRTs for ipsi- and contralateral presentation for all participants. The test was performed on the 2nd degree polynomial detrended times series. The original spectrum (black line) plotted against mean spectrum (blue line averaged across n = 1000 spectral samples from the set of shuffled time series. Red lines indicate one-sided confidence interval bound (). (TIFF) [file pone.0112974.s003.tiff]
