# Supplementary material for: Fronto-Central Theta Oscillations Are Related to Oscillations in Saccadic Response Times (SRT): An EEG and Behavioral Data Analysis
Source: PLoS One. 2014 Nov 18;9(11):e112974. doi: 10.1371/journal.pone.0112974 (PMC4236144; doi:10.1371/journal.pone.0112974)
Supplement: Table S1 — Percentage of errors by type for each participant. (PDF) [file pone.0112974.s009.pdf]

	Participant		
Type of error	1	2	3
Saccades before any signal	0.0	0.0	0.0
Amplitude not within 3 std	0.0	0.9	0.7
SRT $<$ 80	0.9	0.3	0.5
SRT $>$ 500	0.0	0.0	0.0
Directional	1.1	0.2	0.5
Total	2.0	1.4	1.7
