# Supplementary material for: Fronto-Central Theta Oscillations Are Related to Oscillations in Saccadic Response Times (SRT): An EEG and Behavioral Data Analysis
Source: PLoS One. 2014 Nov 18;9(11):e112974. doi: 10.1371/journal.pone.0112974 (PMC4236144; doi:10.1371/journal.pone.0112974)
Supplement: Table S2 — Dependence of frequencies with maximum power on order of detrending polynomial. Frequencies with maximum power after detrending the median data series with polynomials of two different degrees. (PDF) [file pone.0112974.s010.pdf]

Participant	1		2		3	
Presentation condition	ipsi	contra	ipsi	contra	ipsi	contra
Degree of polynomial						
2	7	3	7	6	4	4
5	7	11	6	7	10	7

Frequencies with maximum power after detrending the median data series with polynomials of two different degrees.
