# Supplementary material for: Fronto-Central Theta Oscillations Are Related to Oscillations in Saccadic Response Times (SRT): An EEG and Behavioral Data Analysis
Source: PLoS One. 2014 Nov 18;9(11):e112974. doi: 10.1371/journal.pone.0112974 (PMC4236144; doi:10.1371/journal.pone.0112974)
Supplement: Text S1 — Detrending with different degree polynomials. (PDF) [file pone.0112974.s011.pdf]

Detrending with different degree polynomials

Figure S1 A shows the zeromean difference when detrending the curve of the mean RT with effect of oscillatory activity shown in Figure 2 with a $2nd$ degree polynomial. The black dashed lines have equal distance to the zero line. A trend is still recognizable in the oscillation. Figure S1 B shows the zeromean difference when detrending the same curve with a $5th$ degree polynomial which appears to be more symmetric around the zeromean.
