# Supplementary material for: Fronto-Central Theta Oscillations Are Related to Oscillations in Saccadic Response Times (SRT): An EEG and Behavioral Data Analysis
Source: PLoS One. 2014 Nov 18;9(11):e112974. doi: 10.1371/journal.pone.0112974 (PMC4236144; doi:10.1371/journal.pone.0112974)
Supplement: Text S2 — Analysis on trends. (PDF) [file pone.0112974.s012.pdf]

Analysis on trends

The trend component, $SRT_{Trend}(t)$, which was eliminated from the time series, $SRT(t)$, may itself include slow periodic components in modulating the SRT. To extract them, the trend of the trend function $SRT_{Trend}(t)$ was removed by a polynomial function of degree 2. Again, this was necessary because the long scaled structure of $SRT_{Trend}(t)$ was superimposing small ones and completely nullified the estimation of the frequency spectral content. Furthermore, the polynomial function of degree 2 was chosen since it roughly described the trend and does not represent a periodic structure. Figure S2 shows the procedure, exemplified for participant P3 for ipsilateral stimulus presentation. The frequency with maximum power is identical to the frequency with maximum power when performing the analysis on the original data detrended with only a $2nd$ degree polynomial. This justifies the choice of a higher degree of polynomial for trend removal to be sure that not the overall trend determines the fequency with maximum power. Table S2 shows the frequency with maximum power when applying a $2nd$ and $5th$ degree polynomial for detrending the the behavioral time series. Figures S3 shows the test for the $2nd$ degree polynomial detrended times series.
