# Supplementary material for: Fronto-Central Theta Oscillations Are Related to Oscillations in Saccadic Response Times (SRT): An EEG and Behavioral Data Analysis
Source: PLoS One. 2014 Nov 18;9(11):e112974. doi: 10.1371/journal.pone.0112974 (PMC4236144; doi:10.1371/journal.pone.0112974)
Supplement: Text S3 — Wavelet analysis. (PDF) [file pone.0112974.s013.pdf]

Wavelet analysis

Figure S4-S8 show the results of the EEG analyses when wavelets rather than Hilbert transform is used. While the topographical details of the patterns differ between the Hilbert transform and the wavelet transform, both methods lead to the conclusion that there is a difference in oscillatory phase between the slow and fast SRTs (i.e., peak and trough of the behavioral oscillations), as well as significant phase reset by the first auditory stimulus.
